# Supplementary material for: Afil, a Lectin from Aplysina fistularis, Exhibits Antibiofilm and Synergistic Antibacterial Activity Against Resistant Bacteria
Source: Microorganisms. 2025 Jun 10;13(6):1349. doi: 10.3390/microorganisms13061349 (PMC12195231; doi:10.3390/microorganisms13061349)
Supplement: Supplementary file 1 [file microorganisms-13-01349-s001.zip › microorganisms-3656509-supplementary.pdf]

Supplementary Material

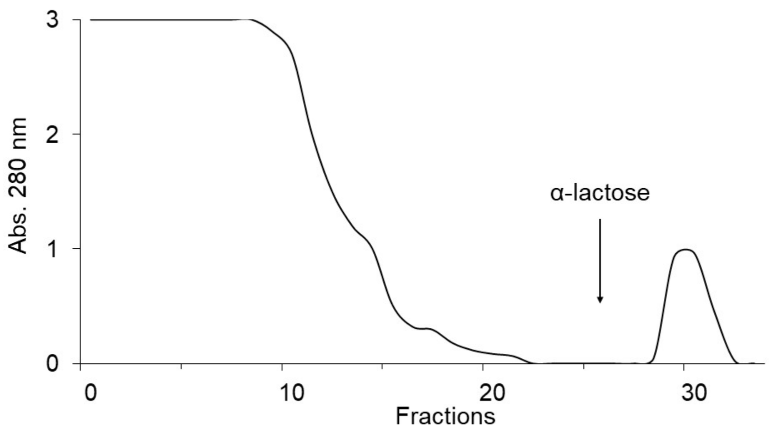

Supplementary Figure S1. Purification of AfiL. Affinity chromatography of the precipitated extract (F 0–70) from *Aplysina fistularis* on a Sepharose 4B column (1.5 × 6.0 cm), previously equilibrated with 50 mM Tris-HCl buffer, pH 7.6, containing 150 mM NaCl (TBS). After unbound proteins were removed, the retained fractions were eluted with 300 mM lactose. The flow rate was maintained at 1.0 mL/min. The arrow indicates the point at which the lactose-containing buffer was introduced. Absorbance was monitored at 280 nm.

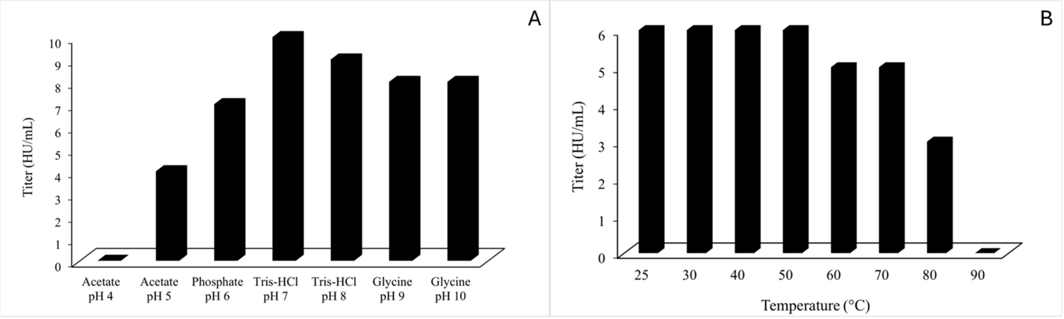

Supplementary Figure S2. Physicochemical properties of AfiL hemagglutinating activity. (A) Effect of pH on the hemagglutinating activity of AfiL. The lectin was incubated in buffers ranging from pH 4.0 to 10.0 prior to assay. (B) Effect of temperature on the hemagglutinating activity of AfiL. The lectin was incubated

at temperatures from 25 °C to 100 °C for 60 minutes before performing the assay. Activity was expressed as the highest dilution showing visible agglutination.

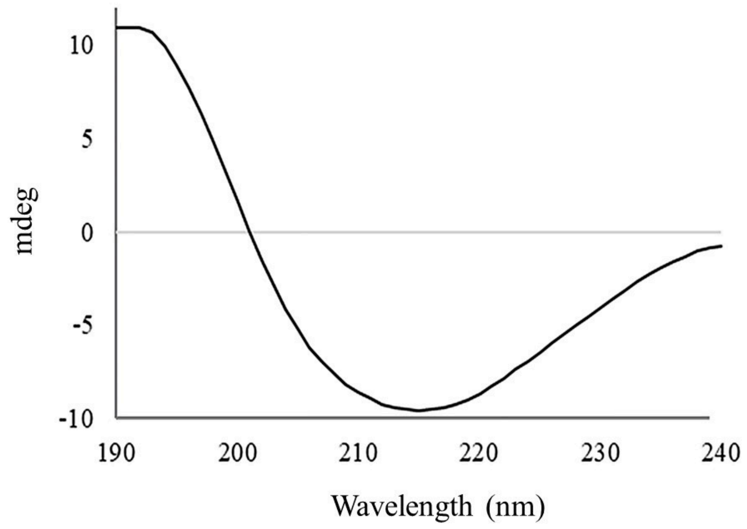

Supplementary Figure S3. Far-UV Circular Dichroism (CD) spectrum of AfiL. CD spectrum of AfiL recorded in the far-UV region (190–240 nm), showing a negative peak at 216 nm indicative of a β-sheet-rich secondary structure. The protein was analyzed at 0.2 mg/mL in 20 mM phosphate buffer (pH 7.0) at 20 °C.

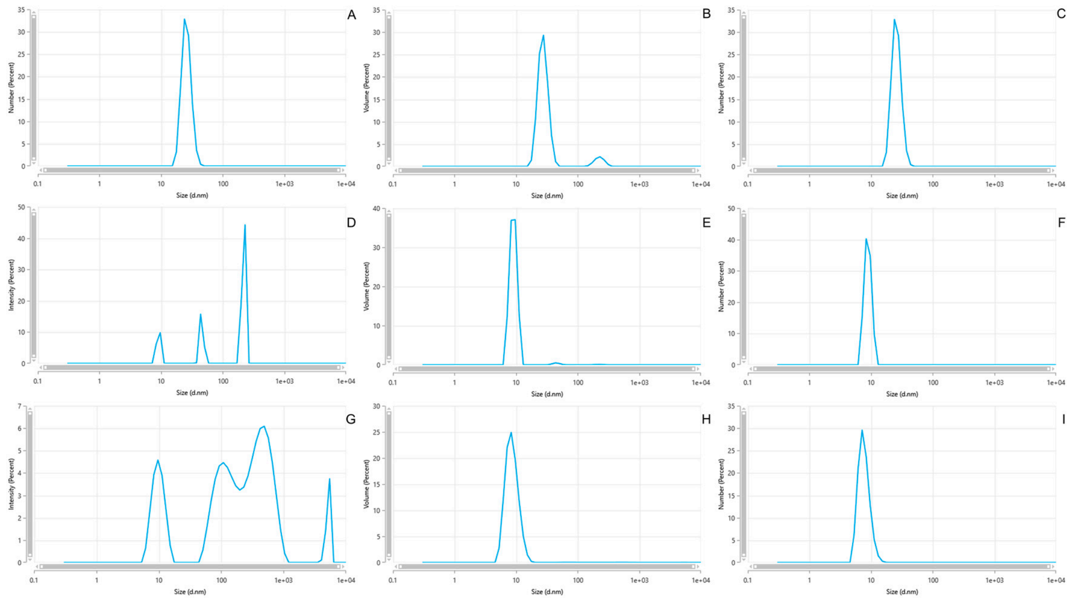

Supplementary Figure S4. Hydrodynamic distribution of AfiL under different pH conditions. Dynamic light scattering (DLS) profiles of AfiL obtained in acetate buffer (pH 5, A–C), phosphate buffer (pH 7, D–F), and glycine buffer (pH 10, G–I). Size distributions were measured based on (A, D, G) intensity, (B, E, H) volume, and (C, F, I) number. Each panel displays the major particle populations detected under the corresponding condition. The intensity-based distribution emphasizes larger aggregates due to the sixth-power dependence of scattering on particle size, whereas volume- and number-based distributions provide a more accurate representation of smaller species. Measurements were performed at 20 °C using a Zetasizer Advance Ultra (Malvern Instruments, UK). The results indicate pH-dependent shifts in particle size and polydispersity, suggesting structural rearrangements and aggregation behavior of AfiL in response to different ionic environments.

Supplementary Table S1. Amino acid sequences of AfiL peptides obtained by MS/MS.

Peptide	*m/z*	Sequence	Mass		Δ
			Observed	Calculated	(Da)
T1	657,17	NYGSPDGFNSQK	1,312,33	1,312.56	0.23
T2	676,23	QHIFSYASGWR	1,350,44	1,350.68	0.24
T3	438,15	LVACPCR	874.34	874.41	0.13
T4	920,32	PVQAFVGDNYYCDSVELASGKVDTK	2,703.96	2,704.26	0.30
T5	921,33	TIDEAYVDGLSITHGSPR	1,929.72	1,929.94	0.22
T6	737,29	HDQIDEAYVDGLSITHGSPR	2,208.87	2,208.95	0.08
T7	681,75	TFLFSYATAWR	1,361.49	1,361.67	0.18
T8	412,6	FDHHLR	823.30	823.40	0.10
T9	902,69	LWFPDTLLWHAATGCSDTDLACR	2,705.04	2,705.30	0.26
Q1	662,74	SAVCGLVEGRNY	1,323.4	1,323.63	0.16
Q2	695,47	PPETIDEAYVDGLSITHGSPRQHIF	2,777.88	2,778.36	0.48
Q3	399,16	PRQHIF	796.32	796.43	0.11
Q4	471,18	GSPRQHIF	940.35	940.39	0.04
Q5	456,67	ANSAGTSTGF	911.32	911.40	0.08

T- peptides obtained by trypsin; Q- peptides obtained by chymotrypsin. m/z: mass/charge. Δ: Cal. Mass -Obs. Mass.
